# Frequency response of ice streams

**DOI:** 10.1098/rspa.2012.0180

**Published:** 2012-06-27

**Authors:** C. Rosie Williams, Richard C. A. Hindmarsh, Robert J. Arthern

**Affiliations:** British Antarctic Survey, High Cross, Madingley Road, Cambridge CB3 0ET, UK

**Keywords:** frequency response, ice stream, ice-shelf buttressing, Fourier analysis

## Abstract

Changes at the grounding line of ice streams have consequences for inland ice dynamics and hence sea level. Despite substantial evidence documenting upstream propagation of frontal change, the mechanisms by which these changes are transmitted inland are not well understood. In this vein, the frequency response of an idealized ice stream to periodic forcing in the downstream strain rate is examined for basally and laterally resisted ice streams using a one-dimensional, linearized membrane stress approximation. This reveals two distinct behavioural branches, which we find to correspond to different mechanisms of upstream velocity and thickness propagation, depending on the forcing frequency. At low frequencies (centennial to millennial periods), slope and thickness covary hundreds of kilometres inland, and the shallow-ice approximation is sufficient to explain upstream propagation, which occurs through changes in grounding-line flow and geometry. At high frequencies (decadal to sub-decadal periods), penetration distances are tens of kilometres; while velocity adjusts rapidly to such forcing, thickness varies little and upstream propagation occurs through the direct transmission of membrane stresses. Propagation properties vary significantly between 29 Antarctic ice streams considered. A square-wave function in frontal stress is explored by summing frequency solutions, simulating some aspects of the dynamical response to sudden ice-shelf change.

## 1. Introduction

The dynamics of the fast-flowing ice streams that drain large ice sheets are key to predicting the future of these ice sheets and their contribution to sea-level change. Changes in the forcing at the ice front are of particular significance, as these affect the position of the grounding line, where ice flow detaches from the bed, which in turn affects the water stored above sea level. For rapidly sliding, water-terminating glaciers, the downstream ice shelf has long been thought to buttress the ice sheet and suppress high-velocity ice flow into the ocean (Hughes 1973). The very low friction at the bed of some ice streams has been thought to facilitate the *instantaneous* long-distance transmission of dynamic stresses (Thomas 2004; Thomas *et al.* 2004), with the implication that velocity fields should respond to downstream changes far upstream. When such dynamical changes result in acceleration, they are presumably precursors to thinning. It is widely accepted that this long-distance instantaneous change operates in ice shelves, and the almost frictionless base of many ice streams is advanced as the reason for supposing this mechanism operates in ice streams. However, the idea of long-distance propagation was an initial *assumption* by Thomas *et al.* (2004) and Thomas (2004), and opposing arguments have been put forward, most recently by Van der Veen *et al.* (2011), who suggested that changes in Jakobshavn Isbræ are predominantly due to ice weakening at the lateral shear margins, based on the notion that long-distance transmission of stresses is damped by lateral and basal resistance. Moreover, a recent theory of dynamics (Schoof 2007), which describes unstable retreat of grounding lines arising from a boundary-layer effect, does not require the long-distance transmission of stresses, which seemingly implies that such transmission is not an essential ingredient of the marine ice-sheet instability. This raises the issue of understanding rapid, long-distance transmission and interpreting the apparent effects seen in observations.

Because there is no universally agreed definition of what constitutes either ‘rapid’ or ‘long distance’, we shall adopt the following convention. Hindmarsh (2012) suggested that there is a frontal mechanical boundary layer in ice streams with frictionless beds, with the longitudinal extent being equal to the width of the stream, over which stresses decay from the front. Stress transmission over distances greater than this will be regarded as long distance in this paper. Hindmarsh (2006*a*) also showed that, for streams with substantial basal resistance, the boundary layer has an extent of 10–20 km, which again defines a minimum scale for ‘long distance’ for basally resisted streams. ‘Rapid’ constitutes effects occurring on time scales faster than the ratio of ice stream length to ice-flow speed.

There is no doubt that rapid upstream propagation of thinning and velocity changes occurs. The events after the collapse of the Larsen A and B ice shelves show this clearly; substantial speed-up of parts of glaciers adjacent to the grounding line was observed almost instantly upon ice-shelf collapse (Rignot *et al.* 2004; Scambos *et al.* 2004) and spread upstream over the following years (Rott *et al.* 2002; Pritchard *et al.* 2011), associated with strong thinning. There are now a large number of similar examples, e.g. Jakobshavn Isbrae (Joughin *et al.* 2008) and glaciers in West Antarctica (Rignot 2006; Pritchard *et al.* 2009). Calving events have been correlated with instantaneous increases in velocity along the length of Helheim Glacier (Nettles *et al.* 2008). Pine Island Glacier (PIG) in the West Antarctica ice sheet (WAIS), which has the largest discharge of all of the WAIS ice streams (Bentley *et al.* 1991), has the potential to contribute significantly to sea-level rise over the coming centuries on account of the reverse bed slope and the recently observed rates of acceleration, thinning and retreat (Rignot 1998; Shepherd *et al.* 2001; Joughin *et al.* 2003; Thomas *et al.* 2004). Because these dynamic changes can be attributed to changes in the conditions at or near the grounding line (Payne *et al.* 2004; Thomas 2004), it is imperative to understand and appropriately model the upstream propagation of these changes when making predictions of sea-level rise for this century and beyond.

Nevertheless, it remains to be conclusively demonstrated that the acceleration of PIG is due *directly* to the transmission of membrane stresses (the three-dimensional version of longitudinal stresses), and some observational evidence exists to the contrary. Joughin *et al.* (2003) found from measurements that changes in driving stress consistent with observed thinning were sufficient to explain much of PIG's upstream acceleration. Scott *et al.* (2009) found that, while acceleration in the grounding-line region is rapidly transmitted upstream on decadal timescales, inland acceleration is correlated with changes in the gravitational driving stress, and that no changes in longitudinal stress gradients were required to explain the changes in velocity.

The observational data thus highlight that upstream propagation of forcing at the ice front can occur through two mechanisms. One is the direct transmission of membrane stresses, acting along the body of the ice stream in the horizontal plane. The other process is through increased flow at the grounding line inducing changes in the geometry of the ice stream, notably steepening, that lead to increases in the gravitational driving stress and velocity. The aim of this paper was to understand the conditions under which both of the two mechanisms operate, and whether a clear distinction can be made between the two.

Our approach is inspired by Nye (1965), who studied the frequency response of glaciers to periodic perturbations of the accumulation or ablation rate in space, for a model based on the shallow-ice approximation (SIA). Here, *frequency response* means quantification of the relationship between spatial scales of response and frequency of forcing. A simple analogy is the way that temperature forcing propagates into a solid, e.g. snow. A forcing with a particular period induces a typical decay length and wavelength in the temperature field. Our fundamental objective is to determine when membrane stresses need to be incorporated into ice-stream models to accurately reflect observations, and how the time scale of the forcing at the ice front affects upstream propagation of velocity and thickness changes.

Shallow-ice models respond to forcing through geometric coupling, and are a well-researched topic in ice dynamics (Hutter 1983). These studies emphasize the fact that the decay time of a perturbation depends monotonically upon its wavelength, with longer wavelength perturbations decaying more slowly, as the slopes are smaller. Accurate representation of long-distance stress transmission requires the incorporation of membrane stresses to model the effects of ice-shelf or frontal changes on inland ice flow. Such stresses are now incorporated in some higher order large-scale ice-sheet models (Blatter 1995; Pattyn 2003; Pattyn *et al.* 2008; Bueler & Brown 2009; Price *et al.* 2011). One scheme for including these stresses in ice-sheet models, the vertically integrated membrane stress approximation (MSA), has proved useful (Kamb & Echelmeyer 1986; Muszynski & Birchfield 1987; MacAyeal 1989; Hindmarsh 2006*a*). Detailed numerical modelling studies of PIG (Payne *et al.* 2004; Dupont & Alley 2005) and Greenlandic glaciers (Nick *et al.* 2009; Price *et al.* 2011) found that the effects of ice-shelf thinning or removal can indeed be rapidly transmitted upstream, increasing velocity and thinning, indicating a strong coupling between surrounding ocean and inland dynamics. However, many large-scale whole ice-sheet models still use the SIA and do not account for upstream propagation of frontal forces and may thus be unable to account for rapid dynamical changes near the ice front (Bamber *et al.* 2007; Vieli & Nick 2011). If there is a class of problems for which membrane stresses are important, then the SIA cannot be used to describe the dynamics in these cases. We therefore aim to provide a deeper insight into the processes that modulate upstream transmission of ocean forcing. A significant outcome is the possibility of assisting in the design of numerical schemes in large-scale ice-sheet models; for example, quantifying the spatial and temporal resolution necessary to capture the dynamics of ice streams and outlet glaciers.

In this study, we use a simplified, vertically integrated, one-dimensional flow-line model of a basally or laterally resisted ice stream, described in §2. This model includes membrane stresses (based on MacAyeal (1989)) and we use it to investigate upstream propagation of a periodic forcing applied to stresses near the grounding line. We apply this forcing at a small distance upstream from the grounding line to avoid having to specify the details of the mechanism. Schoof (2011) found that the errors introduced by the depth integration are small, even close to the grounding line. The use of a periodic forcing allows the model solution to be obtained analytically, which provides direct quantification of the amplitude and phasing effects that varying frontal forcing has on inland thickness and velocity profiles (see §3). These are characterized in terms of exponentially damped waves, with decay length and wavelength that are functions of ice-stream configuration, rheology and frontal forcing period.

Using model parameters from 29 Antarctic ice streams, we characterize the upstream propagation for a range of different forcing frequencies (§4). For each ice stream, we find two distinct types of behaviour dependent on the forcing frequency. Many ice streams have laterally resisted sections abutting basally resisted sections. In §5, a solution is presented for an ice stream that changes from lateral to basal resistance, broadening this methodology by allowing stacking of differently resisted ice-stream portions to better represent real ice-stream conditions. Our linearized model formulation allows the summation of different frequency forcings to create arbitrary forcings close to the ice front. This is demonstrated in §6 through the construction of a square-wave function for the strain rate just upstream of the grounding line, which may provide an approximation of the effects of ice-shelf thinning and thickening at the grounding line on the inland velocity and thickness. The work concludes with a discussion in §7.

## 2. Ice-stream model formulation

The physical basis of our model is similar to that of Payne *et al.* (2004), Dupont & Alley (2005), Walker *et al.* (2008) and Nick *et al.* (2009), except that here we use an idealized ice stream with periodic forcing to allow analytical rather than numerical results to be obtained. We consider a one-dimensional flow-line model for an ice stream of length [X*], such that −[X*]<x*<0 represents the horizontal position. Thickness is given by H*(x*,t*)=s*(x*,t*)−b*(x*), where s*(x*,t*) represents the ice surface and b*(x*) the ice base. Time is denoted t*. The forcing is prescribed at x*=0, which is considered to be a small distance upstream of the grounding line. Grounding-line movement is not modelled explicitly, but we allow thickness changes at x*=0 to implicitly describe such motion. Hence, our focus in this study was on propagation and not on instability. Dimensional quantities are denoted with an asterisk (*) and non-dimensional quantities without.

**Figure 1.**
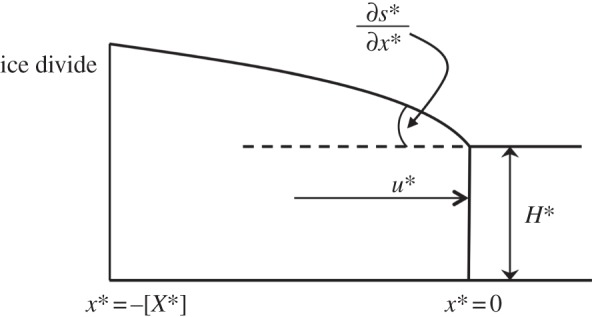
Schematic of the ice-stream model, where u* is velocity along the ice stream and H* is ice thickness. The ice stream flows from the ice divide at x*=−[X*] to x*=0, just upstream of the grounding line. The gradient in surface slope at x*=0 is shown, which is set to *ε* at zeroth order.

We use the vertically integrated ice-stream model of MacAyeal (1989) in one dimension
2.1
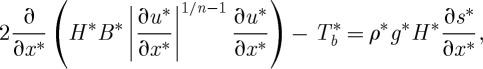
where B* is the ice-stiffness parameter, u* is ice velocity, 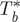 is the frictional resistance we term the traction, ρ* is the density of ice and g* is the gravitational acceleration. Here B^*−*n*^=A*_v_, where A*_v_ is the rate factor in the viscous relationship e*=A*_v_τ^*n^ relating the strain rate e* to the deviator stress invariant τ*. Depth-averaged viscosity for the one-dimensional case was used to obtain the force balance equation (2.1). The first term in equation (2.1) represents the membrane stresses, the second term is the basal traction and the term on the right-hand side is gravity-driven stress. This is often referred to as the ‘shallow stream approximation’ and here we refer to it as the ‘MSA’. If the basal friction term is zero, this represents a shallow shelf approximation, whereas if the membrane stress term is set to zero, it represents the SIA. The driving stress (on the right-hand side of equation (2.1)) plays a role in both approximations. The boundary conditions at the upstream end are that any perturbations die away towards the ice divide, x*=−[X*] (ice thickness at the ice divide can be estimated from a Vialov (1958) profile). The continuity equation is also needed to complete the system,
2.2
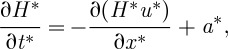
where a* is accumulation. A schematic of the modelled ice stream is shown in figure 1. The exact dependence of the traction 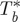 on the velocity depends on whether the ice stream is basally or laterally resisted. Both cases are dealt with in the following sections, in which the model is scaled, a linear perturbation analysis is performed and the linear equations are solved for periodic forcing.

## 3. Frequency response of ice streams

### (a) A basally resisted ice stream

For a basally resisted ice stream 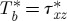 and a sliding law relationship is given by
3.1
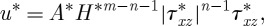
where A* is a coefficient representing either the rate of sliding or the rate of internal deformation, in which case A*=2A*_v_/(*n*+2). *n* is a rheological index and *m* is a constant which is either *n*+2 (for internal deformation according to a nonlinear viscous law) or *n*+1 (for sliding according to a Weertman-type law). The problem is scaled using
3.2
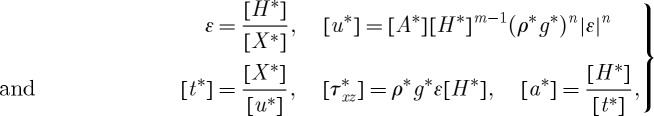
where *ε* is defined as the aspect ratio of the ice stream and length is scaled with the distance from the divide to the grounding line, [X*]. A* and B* are scaled with [A*] and [B*], respectively. The velocity scale [u*] is prescribed and used to calculate the sliding coefficient [A*]. This gives the non-dimensional momentum equation (2.1) as
3.3
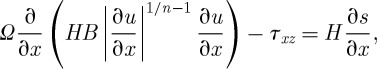
where
3.4
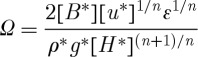
is a dimensionless measure of viscosity and is typically much smaller than unity. The sliding law in equation (3.1) and the continuity equation become
3.5
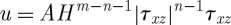
and
3.6
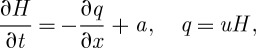
where *q* is ice flux. Now consider a linear perturbation,
3.7
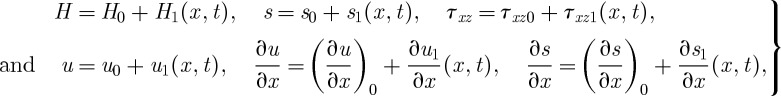
where zeroth-order components (denoted with subscript 0) are constants (*s*_0_=*H*_0_=*u*_0_=*τ*_*xz*0_=1) and first-order components (denoted with subscript 1) depend upon *x* and *t*. In this study, we are predominantly interested in the length scales associated with the upstream propagation of frontal effects occurring near the grounding line. For this reason, we choose the thickness, velocity and strain-rate scales at *x*=0 and define the zeroth-order solution from these parameters. We assume that the negative surface slope at the grounding line is the aspect ratio of ice thickness to ice-stream length, *ε* (e.g. ds*/dx*=−*ε* at zeroth order in figure 1). This choice is compatible with real-world ice streams, and the sensitivity to this assumption is explored in §4. If we define *γ* as the dimensionless zeroth-order uniform strain rate at the grounding line, then, after scaling,
3.8
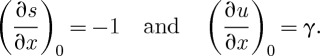
For this choice of zeroth-order slope, *γ*=2 corresponds to steady state (see the electronic supplementary material, §S1). The scaled sliding factor and ice stiffness are not perturbed and are set as constants, *A*=*B*=1 (in dimensional units, A*=[A*], B*=[B*]).

Consider an ice stream such that *b*=0 and *H*_1_(*x*,*t*)=*s*_1_(*x*,*t*). At first order equations (3.3), (3.5) and (3.6) simplify to
3.9
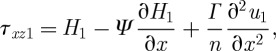

3.10
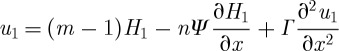

and
3.11
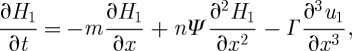
where
3.12
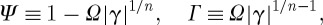
and the nonlinear perturbation terms are dropped. Next, consider a transformation into spectral coefficients,
3.13
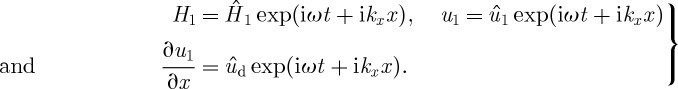
*ω* is the frequency of the frontal forcing (which is restricted to be real), and *k*_*x*_ is a complex spatial wavenumber. This transformation provides the perturbations in terms of frequency responses. Any of the thickness, velocity or strain rate can be chosen as the forcing, with specified amplitude, and the other two perturbation amplitudes are then found by solving the model. We choose to use the strain rate as the leading forcing by setting 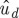. Substituting the spectral transforms into equations (3.9) and (3.10) produces a phasing relationship between the velocity, thickness and strain-rate perturbations depending on *k*_*x*_,
3.14
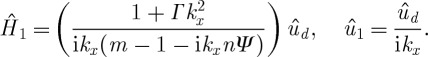
Substituting these transformations and the phasing information into the continuity equation (3.11) leads to a cubic characteristic equation for wavenumber as a function of constants describing ice rheology and periodic forcing,
3.15
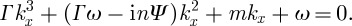
For any given forcing frequency *ω*, solving this cubic for *k*_*x*_ provides estimates of spatial wavelength
3.16
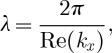
decay length
3.17
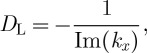
where *D*_L_ is the length upstream at which only *e*^−1^ of the perturbation remains, and the speed of upstream propagation of the resulting perturbation in velocity or thickness (phase speed) is given by
3.18
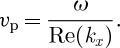
The group velocity is different from the phase velocity, and thus there is dispersion in the system.

### (b) The shallow-ice approximation

Nye (1965) solved a similar problem for the SIA but with frequency variations in the accumulation. The MSA problem can be modified to give the corresponding SIA solution for frontal forcings in the strain rate as follows. The force balance equation is given by equation (2.1) with B*=0 and 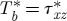 for the basal case, which can be substituted into the sliding law in equation (3.1) to give

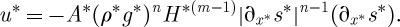
Using the scalings in equation (3.2) along with the assumptions at zeroth order (as discussed above) gives the ice flux as

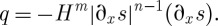
The linear perturbation then results in
3.19
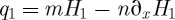
and
3.20
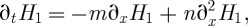
and a transfer to spectral coordinates of the above equation gives a quadratic for *k*_*x*_ as a function of *ω*,
3.21
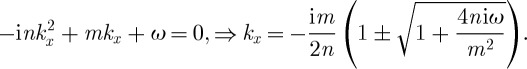
This expression is the equivalent to the cubic equation (3.15) for the MSA as *Ω* tends to zero. Thus, the SIA is the low *Ω* limit of the MSA, corresponding to very weak coupling in the membrane term.

### (c) Limiting behaviour

The high-frequency limit of the frequency response can be calculated from equation (3.15) by expanding in terms of the complex wavenumber *k*_*x*_=Re(*k*_*x*_)+i Im(*k*_*x*_). Analysing the balance of the terms for *ω*≫1 gives equations for the real and imaginary parts of the equation separately as
3.22
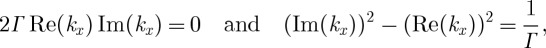
respectively. Thus, 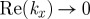 and 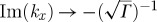 as 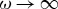 and this provides the spatial wavelength and the minimum decay length *D*_L_ for the MSA and SIA (for which *Γ*=0: see equation (3.12)) as
3.23
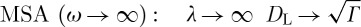
and
3.24
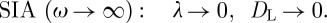
In dimensional units, the limit for the MSA decay length is
3.25
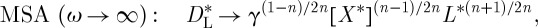
using equation (3.12), where L* is defined as the membrane coupling length (MCL) from Hindmarsh (2006*a*),
3.26
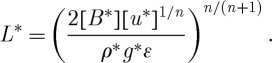
This minimum decay length is not equal to the MCL because it also now depends in part on the length scale of the ice stream and the zeroth-order strain rate at the grounding line. In contrast, for the SIA, *D*_L_ tends to zero for high frequencies. For the MSA in the high-frequency limit, the wave speed diverges (see equation (3.18)).

In the low-frequency limit, the spatial wavenumber and decay number can be calculated in the same manner as for the high-frequency limit (by expanding equation (3.15) and evaluating the terms as 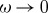). This gives the spatial wavelength and decay lengths in this limit as
3.27
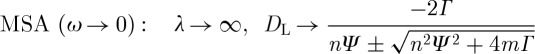
and
3.28
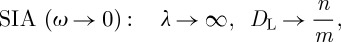
for the MSA and the SIA, respectively, where *D*_L_ is the maximum decay length in this case. The corresponding limits of the wave speed (equation (3.18)) in the low-frequency limit are
3.29
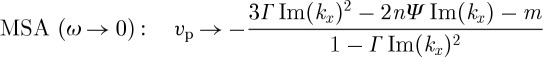
and
3.30
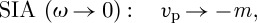
with Im(*k*_*x*_) as 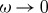 given in equation (3.27).

Equation (3.18) indicates that the speed of upstream propagation (or phase velocity) is dependent on the frequency of the periodic forcing and the spatial wavenumber (which is itself a function of the forcing frequency), and is not directly related to the expected velocity predicted for kinematic wave propagation (Cuffey & Paterson 2010). Even in the low-frequency limit (equation (3.29)), the wave speed for the MSA is more complicated than the kinematic wave speed predicted by the SIA.

### (d) A laterally resisted ice stream

For a laterally resisted ice stream, the lateral drag for a stream of semi-width W* can be solved approximately by using the flow law and writing

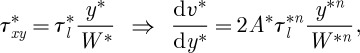
where 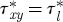 at y*=W*, the lateral margin, and the velocity is v*(x*,y*). Integrating the second of these equations over the semi-width of the ice stream and setting v*=0 at y*=W* gives the centre-line velocity u*(x*)=v*(x*,y*=0) as
3.31
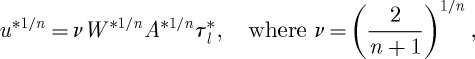
as used by Hindmarsh (2006*a*) (see also Cuffey & Paterson 2010). For the laterally resisted case, A* is the viscous rate coefficient given by A*=B^*−*n*^, and the traction can be written as
3.32
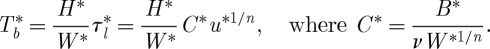
Thus, the horizontal force balance for a laterally resisted ice stream of semi-width W* in equation (2.1) can be written in dimensional units as
3.33
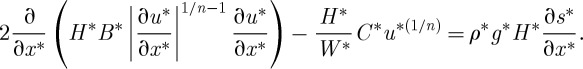
If 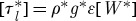 and the same scales for [X*] and [t*] are used as for the basal case, then equation (3.31) gives
3.34
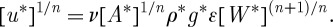
One would usually prescribe the width scale [W*] and then calculate the velocity scale. However, in order to ease comparison between the basal and lateral cases, we choose the velocity scale at the grounding line and derive the width. The zeroth-order slope and strain rate are defined as those of the basal case (see equation (3.8)). The definition of *Ω* is then given by equation (3.4) and the continuity equation (3.6) and scaled momentum equations (3.31) and (3.33) are linearized and transformed into spectral coordinates and solved (see §3*a* and the electronic supplementary material, §S2, for more details). This gives the same phasing relationship (3.14) and the cubic characteristic equation (3.15) as for the basal case, but note that, for the lateral case, *m*=1.

## 4. Results

The cubic equation for basally and laterally resisted streams driven by periodically varying the strain rate at the ice front (3.15) is solved to give the complex wavenumber as a function of the prescribed forcing frequency, *k*_*x*_(*ω*). Perturbations decay towards the ice divide only for Im(*k*_*x*_)≤0. In the case of equation (3.15), our calculations always show one admissible root, although we have not proved this for all cases.

The amplitude of the strain-rate forcing is set to 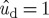 at *x*=0, and because 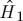 and 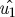 are directly proportional to 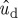, this can be done without loss of generality. Physically, this means we are changing the magnitude of the longitudinal strain rate just upstream of the grounding line, which may be interpreted as, for example, an increase in strain slightly upstream owing to a reduction in back pressure at the grounding line. However, what causes the upstream change in strain rate is not explicitly modelled. Because the basally and laterally resisted cases display the same qualitative behaviour, only a basally resisted example is fully explored (a table of decay length statistics for laterally resisted streams can be found in the electronic supplementary material, §S2 and table S1).

**Figure 2.**
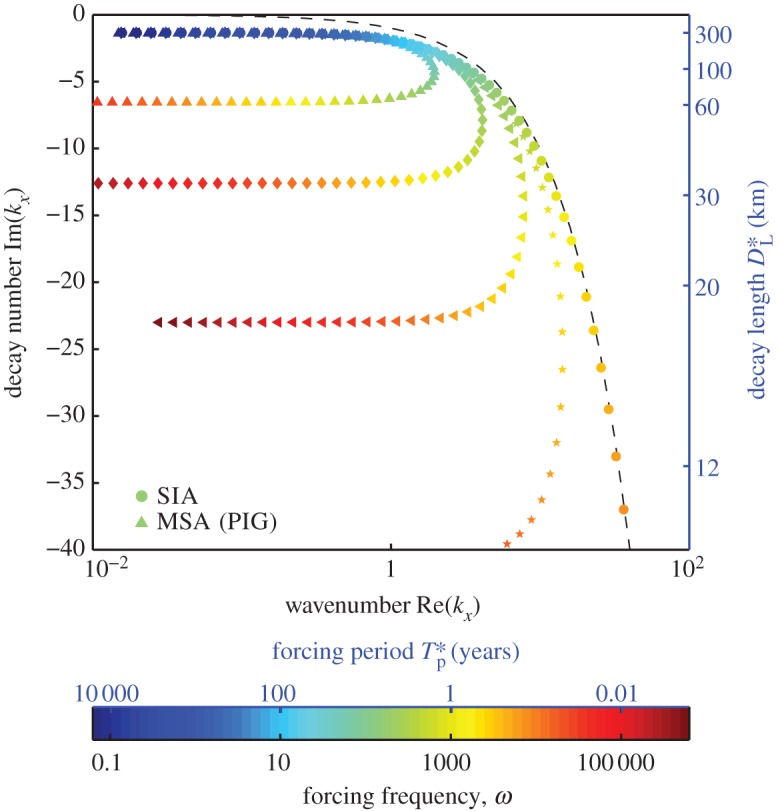
The relationship between frequency *ω* and wavenumber *k*_*x*_ plotted as dimensionless wavenumber (Re(*k*_*x*_)) and decay number (Im(*k*_*x*_)) as a function of forcing frequency *ω* for parameters appropriate to Pine Island Glacier (table 1) for the case of basal resistance. A dimensional scale for the decay length D*_L_ is shown (blue right-hand axis, in kilometres) along with a dimensional scale for the forcing period T*_p_ (top blue axis on colour bar, in years). The SIA is shown as solid circles and the MSA is displayed with a range of *Ω* values, where *Ω*=0.037 is the standard value for PIG (upward pointing triangles). *Ω*=0.01, diamonds; *Ω*=0.003, sideways triangles; *Ω*=0.001, stars. The line on which Re(*k*_*x*_)=−Im(*k*_*x*_) is also shown (black dashed line).

We solve for the complex wavenumber for 29 Antarctic ice streams using drainage basin area and grounding-line velocity and thickness data taken from Rignot *et al.* (2008). This shows how the stream characteristics affect the frequency response. The length scale [X*] is approximated as the square root of the area. The zeroth-order slope is taken to be the aspect ratio of thickness over length scale, as discussed in §3*a*. Although this is an approximation, decay lengths and critical periods were found to be insensitive to variations in *ε*: when *ε* varies by a factor of 10, the minimum decay length for a given ice stream varied by a factor of approximately 3. For this choice of slope, we require *γ*=2 for an ice sheet in steady state at zeroth order (see §3*a* and the electronic supplementary material, §S1). The Glen index *n*=3 is used, and for the basally resisted case *m*=4 (summarized in table 1), whereas, for the laterally resisted case, *m*=1 (summarized in the electronic supplementary material, table S1). We set B*=10^6^ Pa yr^1/3^, corresponding to a temperature of around −30^°^*C*. The dimensionless viscosity parameter *Ω* is small for all streams considered: 0.0066≤*Ω*≤0.037. An example of the relation between forcing frequency and wavenumber, in terms of decay number Im(*k*_*x*_) and spatial wavenumber Re(*k*_*x*_), is shown in figure 2 for the parameters of PIG. Both the MSA (3.15) and the SIA (3.21) solutions are displayed. In this case, the time scale is [t*]=162 years, and the period *T*_p_=2*π*/*ω* is varied between approximately half a day (this corresponds to tidal forcing; note that we are ignoring visco-elastic effects) and 16 000 years (deglaciation time scale).

**Table 1.** Ice streams and outlet glaciers in the Antarctic with parameters taken from Rignot *et al.* (2008), where [H*] and [u*] are grounding-line thickness and velocity, respectively. The length scale [X*] is defined as the square root of the area and we set B*=10^6^ Pa yr^1/3^. *ε* is the ratio of thickness scale to ice-stream length and *Ω* is the dimensionless viscosity parameter. MCL is the membrane coupling length calculated using the expression formulated by Hindmarsh (2006*a*). Decay length D*_L_ and T*_sp_ are calculated using the model of basal resistance, where T*_sp_ is the period at which the spatial wavenumber on the MSA curve of *k*_*x*_(*ω*) changes from increasing as a function of *ω* to decreasing with *ω*, which gives a measure of the demarcation between the fast and slow forcing branches shown in figure 2. Average values for West and East Antarctica are shown. FER, Ferrigno ice stream; PIG, Pine Island glacier; THW, Thwaites glacier; LAN, Land glacier; BIN, Bindschadler ice stream; MAC, MacAyeal ice stream; EVA, Evans ice stream; RUT, Rutford ice stream; INS, Institute ice stream; MOL, Moller ice stream; FOU, Foundation ice stream; SUP, Support Force glacier; REC, Recovery ice stream; SLE, Slessor ice stream; BAI, Bailey ice stream; DAV, David glacier; REN, Rennick glacier; NIN, Ninnis glacier; MER, Mertz glacier; DIB, Dibble glacier; FRO, Frost glacier; TOT, Totten glacier; DEN, Denman glacier; LAM, Lambert glacier; RAY, Rayner and Thyer glaciers; SHI, Shirase glacier; JUT, Jutulstraumen; BYR, Byrd glacier; STA, Stancomb–Wills glacier.

								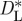 (km)			
							T*_p_=1 (years)		T*_p_=100 (years)		
name	[H*] (km)	[u*] (km yr^−1^)	[X*] (km)	*ε*	*Ω*	MCL (km)	MSA	SIA	MSA	SIA	T*_sp_(years)
FER	1.5	1.7	118	0.013	0.036	9.79	18.3	12.8	69.2	71.3	6.37
PIG	1.1	2.5	405	0.0027	0.037	34.2	62.2	29.6	189	197	15.3
THW	1.1	2	427	0.0026	0.034	33.6	62.5	27.3	183	191	18.3
LAN	1.3	1	114	0.011	0.035	9.28	17.2	9.83	59.1	61.1	10.2
BIN	0.6	0.3	374	0.0016	0.034	29.9	55.1	10.2	80.6	86.8	109
MAC	0.6	0.3	418	0.0014	0.033	32.5	60.5	10.8	86.3	92.6	117
EVA	1.5	0.6	330	0.0045	0.018	16.3	35.3	13.4	101	106	24.4
RUT	2	0.4	230	0.0087	0.013	9.05	21.2	9.13	70	72.7	18.6
INS	1.3	0.4	386	0.0034	0.017	18.4	40.3	11.9	93.2	99.5	40.9
MOL	1.1	0.1	249	0.0044	0.015	10.6	24.1	4.81	39.3	43	90.4
FOU	2.3	0.6	718	0.0032	0.009	21.2	54.4	19.9	161	169	26.1
West:	1.3	0.9	343	0.0051	0.026	20.4	41	14.5	103	108	41
SUP	1.6	0.1	365	0.0044	0.009	10.7	27.5	5.84	48.3	53.1	78.9
REC	1.8	0.8	998	0.0018	0.011	35	84.9	27.1	218	231	34.5
SLE	1.3	0.5	706	0.0018	0.015	30.6	69.2	18.1	144	156	52.4
BAI	2	0.2	266	0.0075	0.01	8.49	21.3	7.01	56.7	60.1	32.3
DAV	2.7	0.5	463	0.0058	0.008	12.9	33.9	14.5	117	121	18.7
REN	1.5	0.2	230	0.0065	0.014	9.44	21.7	6.51	51.7	55.2	39.5
NIN	1.5	0.8	453	0.0033	0.018	22.2	48.2	18.1	137	144	24.9
MER	1.8	0.8	286	0.0063	0.017	13.7	30.1	14.3	104	108	15.2
DIB	1.5	0.8	182	0.0083	0.024	11.2	22.6	11.3	77.1	79.6	13.7
FRO	2	1.7	369	0.0054	0.019	18.5	40.3	23.4	160	164	9.84
TOT	2	0.8	755	0.0026	0.011	26.2	63.9	23.5	187	196	25.8
DEN	2.5	1.5	475	0.0053	0.013	18.3	43.6	25.2	182	186	10
LAM	3	0.7	978	0.0031	0.007	22.7	63.4	25.1	207	216	22
RAY	1	1	322	0.0031	0.032	24.6	46.2	16.9	120	126	26.4
SHI	1.3	2.2	446	0.0029	0.029	31.4	60.7	29.3	196	203	14.8
JUT	2	0.7	351	0.0057	0.014	14.3	33.1	14.9	113	117	17
STA	1.4	0.7	329	0.0043	0.02	17.8	37.4	14.4	107	112	23.6
BYR	2	0.8	998	0.002	0.01	32.3	80.6	27.1	219	232	31
East:	1.83	0.822	499	0.0045	0.016	20	46	17.9	136	142	27.3

**Figure 3.**
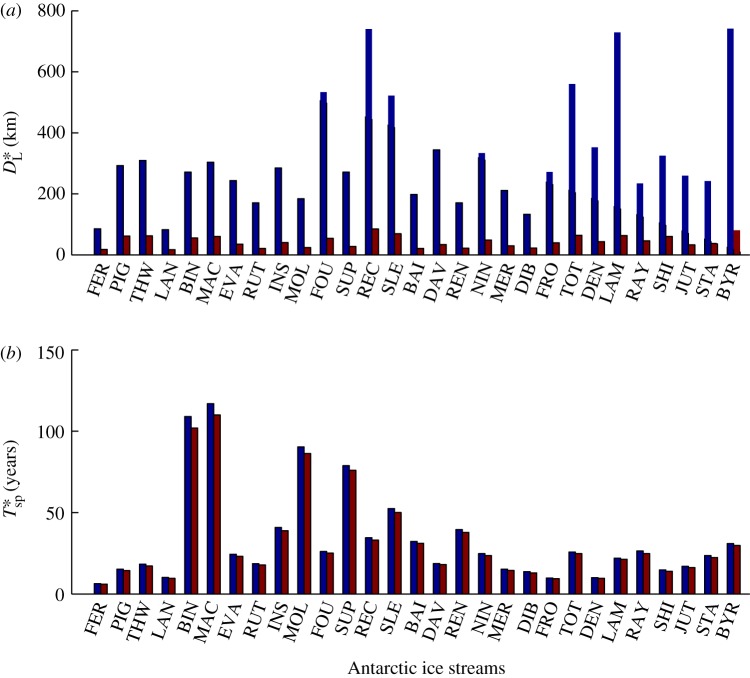
Plots of (*a*) the maximum (blue) and minimum (red) decay lengths D*_L_ for the case of basal resistance and (*b*) the demarcation period between the fast and slow branches T*_sp_ for basal (blue) and lateral (red) resistance, shown for 29 Antarctic ice streams (see the electronic supplementary material, tables S1 and S2, for full data).

Figure 2 is a plot (Argand diagram) of the real and imaginary parts of the wavenumber for different forcing frequency *ω*. It is best to view *ω* as the independent variable, and then plot the relationship between the decay number and wavenumber. The main axes are dimensionless but a dimensional scale for the decay length (kilometres, right-hand axis, blue) and for the colour scale in terms of the dimensional forcing period 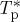 (in years) is also shown for ease of interpretation. Two distinct branches of the curve, both with almost constant decay length, can be distinguished for the MSA using standard PIG parameters (shown as upward-pointing triangles): a slow, low-frequency forcing with a maximum decay length of approximately 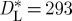 km (Im(*k*_*x*_)=−1.38) and a fast, high-frequency forcing with a shorter minimum decay length of D*_L_=61.9 km (Im(*k*_*x*_)=−6.54). The spatial wavenumber increases with frequency on the upper branch, and decreases as the frequency becomes very high on the lower branch. This dual branch behaviour is a generic feature of the solution, and is seen for all Antarctic ice streams considered in this study. Figure 3*a* shows that the difference between the decay lengths for the slow and fast branches (the maximum and minimum decay lengths, respectively) varies between the ice streams owing to the different geometries and properties of each stream (see also table 1 and the electronic supplementary material, table S2 and §S2). The minimum decay length is independent of *m* and thus is the same for both basal and lateral resistance, varying between 17 and 84.8 km for the 29 ice streams in table 1 (note: 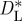 at T*_p_=1 year in table 1 is very close to the minimum decay length). Thus, we find that for all ice streams considered even sub-decadal to decadal forcings can be transmitted tens of kilometres inland. The maximum decay length is around three to four times bigger for the laterally resisted as opposed to basally resisted streams, because the low-frequency limit is *m*-dependent (see equation (3.27)).

The black dashed line in figure 2 shows the line where −Im(*k*_*x*_)=Re(*k*_*x*_) (which, for large *ω*, is approximately the same as the SIA (solid circles)). Complex wavenumbers to the left of this line have wavelength *λ* greater than decay length *D*_L_. It can be seen that for the MSA this can be expressed as *λ*>*D*_L_; in other words, we do not expect to be able to observe the upstream sinusoidal variations except at sufficiently low frequencies, when the spatial wavelength is not significantly greater than the decay length.

**Figure 4.**
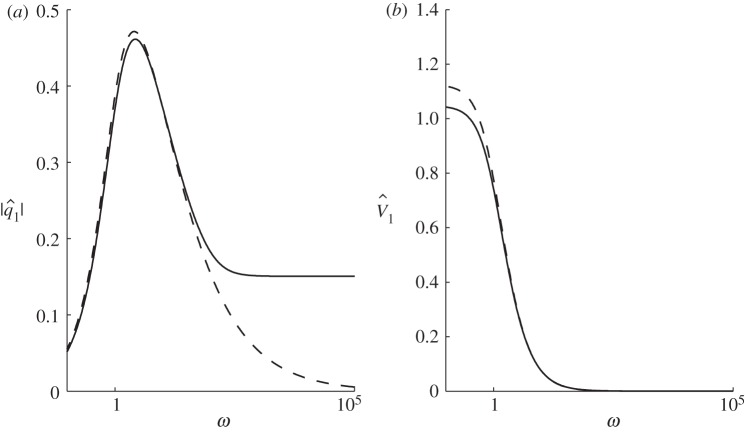
The amplitude of (*a*) the first-order flux perturbation *q*_1_ and (*b*) the first-order integrated flux (or ‘volume’) perturbation (equation (4.1)). Solid lines, MSA; dashed lines, SIA.

Figure 4 shows the frequency response of PIG in terms of the amplitude of the linearized flux perturbation and the amplitude of the integrated flux
4.1
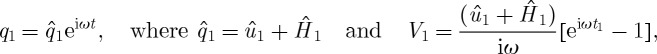
respectively. *V*_1_ is the change in perturbed volume between *t*=0 and some time *t*=*t*_1_ and the amplitude of this volume change over a full period gives the maximum perturbed volume change at some point *t*_1_=*t*_max_, 0≤*t*_max_≤*T*_p_, where *T*_p_ is the dimensionless period. However, because the forcing is periodic, *V*_1_=0 when *t*=0 and *t*_1_=*T*_p_; thus, there is no net change in perturbed volume over one full period. Figure 4 shows that, for slow forcings (when *ω* is small), the magnitude of the flux perturbation is small whereas the maximum perturbed ‘volume’ amplitude is large, presumably because the small flux perturbation acts over a long time period for these slow forcings. On the high-frequency branch (when *ω* is large), the flux perturbation becomes independent of frequency and 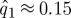. Summed volumetric changes within a high-frequency oscillation cycle are very small owing to the limited time the flux has to build up for short periods. In between these scenarios, the maximum flux amplitude considered as a function of periodicity occurs at *ω*≈2.6 (T*_p_≈388 years).

**Figure 5.**
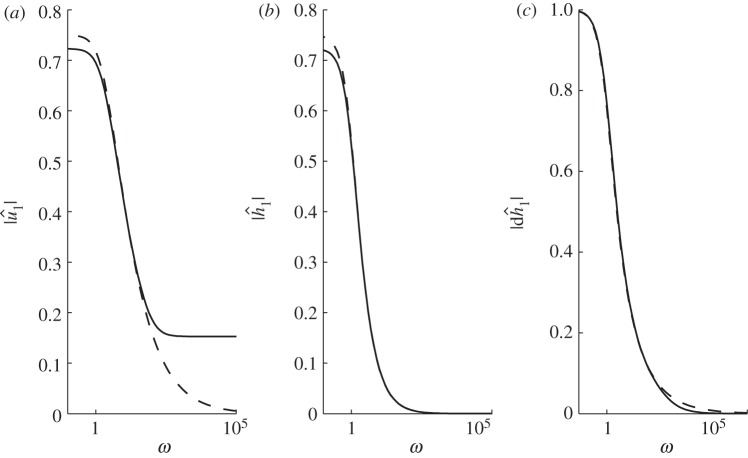
The magnitude of the perturbations in (*a*) velocity, (*b*) thickness and (*c*) positive surface slope, as functions of the frequency of the frontal forcing in strain rate for the case of basal resistance using PIG parameters. Solid lines, MSA; dashed lines, SIA.

**Figure 6.**
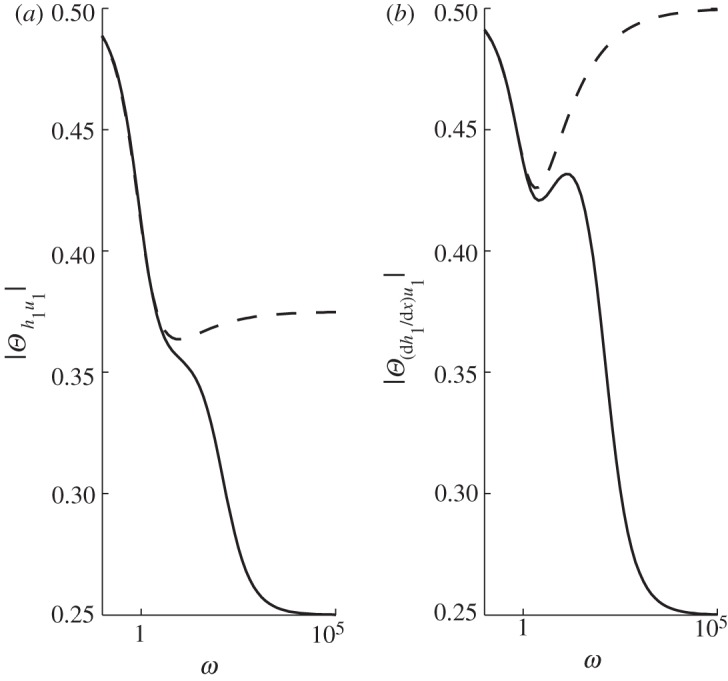
The relative phase angle, normalized with 2*π*, between perturbed (*a*) thickness and velocity, and (*b*) positive slope and velocity as functions of the frequency of the frontal forcing in strain rate for the case of basal resistance using PIG parameters. In-phase/anti-phase is at *Θ*=1,0.5 and *Θ*=0.25/0.75 represents completely out-of-phase behaviour. Solid lines, MSA; dashed lines, SIA.

To understand these perturbations in flux for the MSA, we examine the magnitude of the perturbations in velocity, thickness and slope (d*H*/d*x*) (figure 5) and the phase angle between velocity and thickness and between velocity and slope (figure 6), all as functions of forcing frequency. The phase angles shown in figure 6 are normalized with 2*π*, so that when *Θ*=1 the variables vary in perfect phase and when *Θ*=0.5 they are in anti-phase (i.e. the maximum velocity occurs at the same point as the minimum thickness, for example). At *Θ*=0.25/0.75 variables vary completely out of phase. When the frequency *ω* is low, figure 5 shows that the magnitude of the perturbations in velocity and thickness are substantial. However, figure 6*a* shows that these changes are in almost perfect anti-phase (for 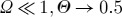). Thus any increase in velocity is compensated by a decrease in thickness, leading to a small flux perturbation (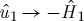 in equation (4.1), thus 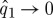). As *ω* increases velocity and thickness perturbations become out of phase but are still of substantial magnitude (e.g. at *ω*=5, 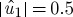, |*Θ*_*h*_1_*u*_1__|=0.36), leading to the maximum flux at *ω*≈2.6. For high-frequency forcings, *ω*≫1, the magnitude of the thickness perturbation becomes very small but the magnitude of the perturbed velocity tends to a limiting value (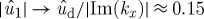) as shown in figure 5*b* and figure 5*a*, respectively. This explains the constant amplitude of the flux for high *ω*, 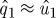, because 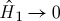 (see equation (4.1)). Thus, in this case, velocity adjusts rapidly to changes in the frontal forcing but thickness does not. Furthermore, velocity moves out of phase with both positive slope and thickness for *ω*≫1, as shown by 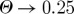 in figure 6*a*,*b*.

**Figure 7.**
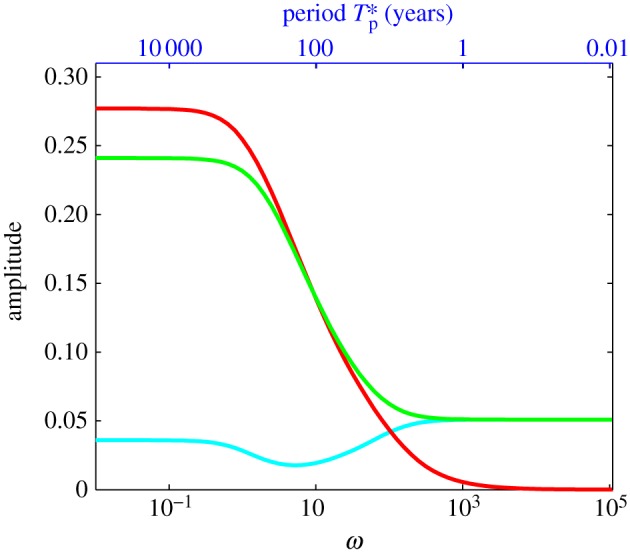
The perturbation amplitude of each term in the force balance (equation (3.3)) as a function of the forcing frequency for the case of basal resistance for PIG parameters. The corresponding dimensional axis for the forcing period T*_p_ is also shown (top axis, blue). Light blue line denotes membrane term; red line, drive term; green line, drag term.

In figure 7, the amplitudes of the perturbed, first-order membrane stress, driving stress and drag terms in the force balance equation (equation (3.3)) are plotted separately as functions of forcing frequency, *ω* (also shown as a function of dimensional period 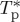 in blue). Note that, owing to variations in phasing with changes in *ω* (shown in figure 6), these amplitudes cannot be directly summed to zero for force balance. At zeroth order, we have steady state, and the drag balances the driving stress. The membrane term is of order *Ω*, and thus appears at first order. In the low-frequency limit (centennial to millennial periods), both the perturbed drag and the driving stress are larger than the membrane stress perturbation and appear to be approximately in balance. On the branch of high frequencies (decadal to sub-decadal forcing periods), the perturbed driving stresses are very small, owing to very little thickness or slope change (figure 5*b*,*c*), and here the perturbed drag and membrane stress terms approximately balance.

For very low frequencies, MSA and SIA predictions of decay length are very similar but not identical (see equations (3.27) and (3.28) and figure 2): for PIG, the maximum decay lengths are within 4 per cent. Figure 2 shows that SIA and MSA predictions of decay number and wavenumber diverge as the forcing frequency increases. Whereas the MSA decay length forms a second branch of asymptoting decay number (D*_L_=62 km, Im(*k*_*x*_)=−6.54), the SIA shows monotone behaviour: as *ω* increases and the forcing becomes rapid, the decay length becomes very small and perturbations decay rapidly as they travel upstream (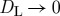 as 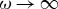; see equation (3.24)). Additionally, the spatial wavenumber for the MSA decreases for high *ω*, while for the SIA the relationship between spatial wavenumber and decay number is linear in the high-frequency limit (see the dashed line in figure 2). These differences in behaviour of the MSA and the SIA for large *ω* account for the differences shown between the approximations in figures 4–6. The forcing period at which the spatial wavenumber on the MSA curve changes from increasing as a function of *ω* to decreasing as a function of *ω* (i.e. when the MSA curve in *k*_*x*_(*ω*) in figure 2 turns back on itself) we denote T*_sp_ and record in table 1. This provides a measure of the demarcation between the fast and slow branches for the MSA, and thus gives an approximate range of forcings 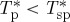 for which the SIA does not capture the dynamics of upstream propagation (since the SIA does not predict significant upstream propagation on the fast branch). For the majority of the ice streams evaluated, this timescale is approximately decadal to sub-decadal (the average for all 29 ice streams is T*_sp_=32.5 years), but varies widely between different ice streams. For example, for PIG T*_sp_=15.3 years (*ω*=0.025) but for the MacAyeal ice stream T*_sp_=117 years, indicating that a forcing of, for example, 50 years may be on the slow branch for PIG but on the fast branch for the MacAyeal ice stream. Similar results are found for laterally resisted ice streams, with slightly smaller T*_sp_ values (see figure 3*b* and the electronic supplementary material, table S1 and §S2). Finally, figure 2 indicates that decay length increases as a function of dimensionless viscosity *Ω*, and for small *Ω* (shown as sideways triangles and stars) the propagations are heavily damped. This can be understood by noting that, as 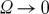, the MSA model becomes the same as the SIA, as exemplified by equation (3.21): the SIA is the low *Ω* limit of the MSA.

## 5. Varying resistance conditions along an ice stream

The stress balance of PIG consists of a 40–50 km region slightly inland of the grounding line with high driving stress balanced by high basal traction, with regions upstream and downstream with much lower driving stress in which longitudinal and lateral stresses play a significant role (Vieli & Payne 2003; Payne *et al.* 2004). To understand this further, we join a downstream laterally resisted region to an upstream basally resisted section. This is achieved by matching the full triple root solution of the lateral problem in the downstream sector to a basally resisted solution in the upstream sector. We prescribe the total forcing just upstream from the grounding line and enforce continuous velocity, thickness and strain rate across the lateral-to-basal transition at *x*=*X*_*C*_, along with the upstream boundary condition that the perturbations tend to zero towards the ice divide (for full details, see the electronic supplementary material, §S3). To preserve scales for the matching at *x*=*X*_*C*_, all parameters and *ω* on both sides of the divide are kept the same; the only difference between the basal and lateral cases is the value of *m* (*m*=1 for the lateral case and *m*=4 for the basal case). Because *m* is not involved in the high-temporal-frequency limit (see equation (3.23)), only low-frequency forcings with periods of decades or longer are affected by the change in resistance.

**Figure 8.**
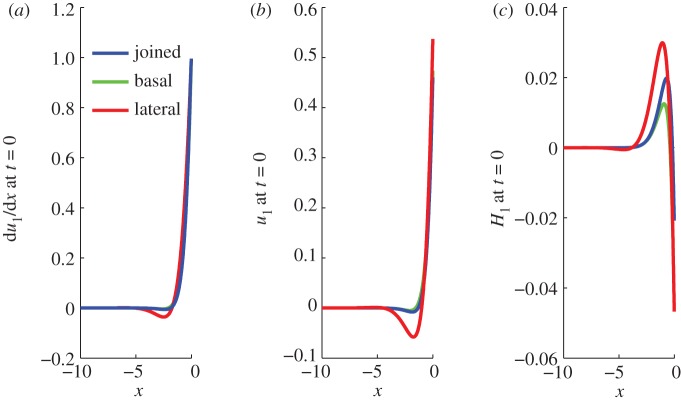
Profiles at *t*=0 of strain-rate, velocity and thickness perturbations along the ice stream for PIG parameters (table 1), where a lateral solution is joined to a basal solution 20 km (0.05 in dimensionless units) from the ice front (blue). The period of the forcing is 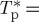
100 years. A purely basally resisted solution (green) and a purely laterally resisted solution (red) are also shown.

An example of an ice stream with parameters estimated for PIG (table 1) is shown in figure 8, where the change from lateral to basal resistance is 20 km upstream of the grounding line (as suggested in Vieli & Payne (2003)) for a period of 100 years. The purely basal and purely lateral cases are also shown here, and because in this case the stream changes to basal resistance close to the ice front, the matched profile is very similar to the basally resisted case. The degree of agreement will vary with the position of the join along the stream. This approach adds flexibility to the method since it can easily be expanded to stack together many ice-stream portions with differing basal conditions to better model the traction of a real ice stream.

## 6. Constructing arbitrary periodic forcings in strain rate

**Figure 9.**
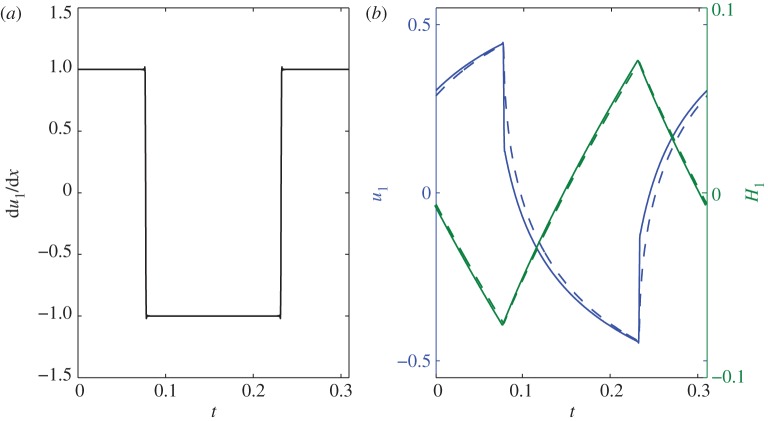
(*a*) A square-wave perturbation in strain rate at *x*=0 with a period of 50 years and (*b*) the corresponding velocity and thickness perturbations for PIG parameters (table 1), where *j*=301. Solid lines, MSA; dashed lines, SIA.

Owing to the linear nature of the perturbation model, multiple solutions can be summed to build an arbitrary periodic forcing close to the ice front. As a simple example, we construct a square-wave function (figure 9) in strain rate at *x*=0, just upstream from the grounding line, with amplitude 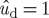 and period *T*_p_,

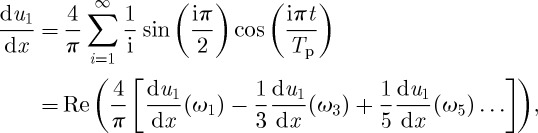
where the frequency is set to *ω*=2*π*(2*i*−1)/*T*_p_, *i*=1,2…,*j*. The step change in strain rate may represent some aspects of upstream changes caused by sudden ice-shelf loss due to, for example, calving events or ungrounding from a pinning point. An example of this square-wave forcing is shown in figure 9 for a period of 50 years, T*_p_=50 years. A step decrease in strain rate leads to a rapid drop in velocity and a slower increase in thickness. The relatively high frequency of the forcing accounts for the small change in thickness and the difference in response speed of thickness and velocity (velocity and thickness are out of phase), as shown in §4 for sinusoidal forcings. Although the periodic square wave is an idealized forcing, the approximate 5–10% thinning and the 50 per cent acceleration of PIG over a period of 25 years shown in figure 9 are of a comparable magnitude to observed changes (Rignot 2008; Pritchard *et al.* 2012). A similar approach could be used to reconstruct more realistic forcings.

## 7. Discussion

We have developed an analytical theory for the upstream propagation of a periodic forcing in the strain rate just upstream of the grounding line. Physically, this change in strain rate could be a response to changes in the back-pressure at the grounding line, perhaps due to ice-shelf thinning or thickening. The fundamental result of this theory is that there are two styles of upstream propagation of velocity perturbations, each associated with different periods of forcings. For low-frequency forcing, the propagation does not depend on membrane stresses while for high-frequency forcing it does.

The model was employed to estimate response characteristics for 29 Antarctic ice streams using grounding-line parameters from Rignot *et al.* (2008). The equivalent ice-stream propagation problem was also solved using the SIA (based on methods by Nye (1965)). Ice streams with varying traction regimes in different regions, such as PIG (Vieli & Payne 2003), were also explored by constructing a method to match basally and laterally resisted ice-stream sections. For both basally and laterally resisted cases, decay length and the spatial wavelength of the upstream perturbations can be characterized as functions of ice rheology, geometry and forcing frequency. High frequencies could represent seasonal or even tidal forcings, although caution is required since tidal forcings are usually modelled (visco-)elastically (Reeh *et al.* 2003; Gudmundsson 2007). Low frequencies could represent some aspects of natural modes of climate variability such as the Atlantic multi-decadal oscillation (as suggested by Price *et al.* (2011)). More general forcing can be constructed from combinations of Fourier components, as demonstrated by the construction of a square-wave forcing. Results for PIG parameters were analysed in detail as an example. We caution that the problem is ultimately nonlinear and large-amplitude response may require further investigation.

For all ice streams considered, as the forcing frequency varies, two distinct response styles emerge: a slow, low-frequency branch (centennial to millennial periods) with spatial wavenumber increasing with frequency, and a fast, high-frequency branch (decadal and sub-decadal periods) with spatial wavenumber decreasing with frequency. On the low-frequency branch, the response can propagate many hundreds of kilometres upstream. On this branch, velocity is approximately in phase with negative slope and in anti-phase with thickness, and drag and driving stress are approximately in balance and both are much larger than the contribution from membrane stresses. Thus, the SIA is sufficient to explain and model the dynamics of the low-frequency branch. The mechanism for propagation on this branch is due to changes in grounding-line flow and geometry rather than direct propagation of membrane stresses. On the high-frequency branch for the MSA, velocity responds very rapidly to forcing, but thickness and driving stress vary little. On this branch, changes in membrane stresses are balanced by changes in drag and the effects are directly propagated tens of kilometres upstream. Thus, we find that there is a clear distinction, based on the frequency of the frontal forcing, between the two mechanisms for upstream propagation. Furthermore, this distinction, in terms of the period at the demarcation between the two branches, varies significantly between ice streams. A surprising feature of the results was the propagation of high-frequency velocity effects beyond the boundary layer lengths proposed by Hindmarsh (2006*a*) and Schoof (2007), for example. This boundary layer length is an appropriate description for static situations, but is not particularly informative about length scales of high-frequency upstream forcing. The difference between the two is conditioned by the nonlinear rheology of ice, as shown by equation (3.25). It should be clear that there is a difference between our fast mode of propagation and ‘instantaneous’ changes transmitted seismically. The details of the relationships between the two waves at high frequency are likely to be complex.

Inland acceleration and thinning have been observed in Greenland and the Antarctic and various theories have been proposed to explain the mechanisms behind these changes. Both Joughin *et al.* (2003) and Scott *et al.* (2009) (for PIG) and Van der Veen *et al.* (2011) (for Jakobshavn Isbræ) found from observations and a force balance analysis that changes in the transmission of longitudinal stresses were not necessary to explain upstream acceleration. Although our study has only dealt with periodic forcings, we would expect some of the results to be more generally applicable. PIG has now been thinning for at least two decades (Shepherd *et al.* 2001). This, together with our findings and those of Joughin *et al.* (2003) and Scott *et al.* (2009), are all consistent with behaviour on the low-frequency branch forced by changes at or near the grounding line. This interpretation is also consistent with the diffusive response modelled by Payne *et al.* (2004). Furthermore, rapid forcing on the high-frequency branch has been observed in the form of rapid seasonal speed-up on Jakobshavn caused by changes in back-stress and ice-front position (Joughin *et al.* 2008). We have clarified the distinction between our upstream propagating waves and kinematic waves in ice streams (Bindschadler 1997; Payne *et al.* 2004, Nick *et al.* 2009); in particular, we find that the upstream propagation rate is frequency dependent, as is the case for downstream flows with non-SIA mechanics (see also Gudmundsson 2003). Observations by Van der Veen *et al.* (2011), of significant changes in driving stress and drag over time which were not accompanied by any significant change in the membrane stress contribution, are consistent with our results provided that the forcing varied sufficiently slowly to keep the dynamical behaviour on the low-frequency branch (figure 7). Thus, whereas Van der Veen *et al.* (2011) attribute the speed-up of Jakobshavn to a weakening of ice at the lateral margins, we find this could be caused by a frontal perturbation. However, this still leaves the surprisingly large magnitude of the speed-up unexplained if the Glen index of *n*=3 is used (higher *n* might explain the magnitude). This highlights the difficulty in attributing any observed changes in thickness or velocity either to a frontal forcing or to spatial anomalies in basal slipperiness or changes at the lateral margins; moreover, it is clear that the frequency of forcing is an important parameter when attempting to make this distinction.

While our results are presented in terms of upstream propagation from grounding lines, we expect the same pattern to hold for variability generated internally in ice sheets (although further investigation is needed to verify this). An example would be penetration of melt to the bed in the ablation zones of thick ice sheets that results in rapid speed-up. This mechanism, long known from Alpine glaciers (Iken *et al.* 1983; Iken & Bindschadler 1986), was observed in Greenland by Zwally *et al.* (2002). Price *et al.* (2008) argued that the forcing need not be local, and could be transmitted long distances through longitudinal stress coupling. Our studies indicate that the frequency of forcing can significantly affect the signal. When coupled with recent work on how the rate of melt supply controls velocity perturbations (Schoof 2010; Sundal *et al.* 2011), we can see that potentially a very complex picture might emerge through further studying such systems.

Although we find that long-distance short-period upstream propagations are not associated with significant changes in thickness, further investigation is required to assess the real-world implications for rapid but non-periodic forcing, and for a nonlinear system. Within a decade, we will have a good forcing and thinning record for most ice streams. Our results clearly imply that each ice stream requires a separate analysis to discriminate between high-frequency and low-frequency forcing, because we find that the upstream response to a forcing is not a simple function of the temporal nature of forcing. The forcing time scale separating the fast and slow branches for each ice stream (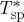) appears to be approximately comparable to the current length of the observational record. These findings agree with data presented by Moon *et al.* (2012), who found that Greenland's outlet glaciers display a complex response to both regional and local forcing over annual to decadal time scales. The problem presented herein is then of potential use if viewed as an inverse problem: if the inland velocity and thickness changes of an ice stream are known, we might hope that the problem can be inverted to provide the temporal forcing at the ice front that caused these profiles. If this problem could be solved, then past changes in ice-shelf forcing owing to calving, fracture or collapse may be inferred, potentially allowing estimation of past ice–ocean interactions that are otherwise difficult to quantify.

Although the SIA represents a low *Ω* limit of the MSA and captures upstream propagation for sufficiently slow forcings, we caution that this does not imply that the SIA is appropriate for modelling ice streams. In particular, it performs poorly at modelling the propagation of decadal and sub-decadal components of the forcing. In addition, we prescribed the strain rate at the grounding-line, but Pattyn *et al.* (2012) have shown that the SIA would not calculate accurate grounding-line dynamics unless augmented by a flux parametrization such as that proposed by Schoof (2007). Furthermore, the SIA has been shown to be ill-posed for some thermo-viscous calculations (Hindmarsh 2004, 2006*b*). We believe that it is inadvisable to use the SIA to model upstream propagation dynamics in ice streams, especially if information about decadal changes is sought.
